# Stimulated generation of photobiogas by morphologically tuned nanostructured ZnO and ZnO/TiO_2_

**DOI:** 10.1186/s13065-022-00866-2

**Published:** 2022-10-03

**Authors:** Omar Mbrouk, H. Hafez, Sylwia Mozia, A. M. Othman, M. S. A. Abdel Mottaleb

**Affiliations:** 1 Nano-Photochemistry and Its Environmental Applications Laboratory, Environmental Studies and Research Institute (ESRI), University of Sadat City (USC), Sadat City, 23897 Menofia Egypt; 2 Department of Inorganic Chemical Technology and Environment Engineering, West Pomeranian University of Technology in Szczecin, ul. Pulaskiego 10, 70-322 Szczecin, Poland; 3 Genetic Engineering and Biotechnology Research Institute, University of Sadat City (USC), Sadat City, Menofia Egypt; 4 Nano-Photochemistry and Solar Chemistry Laboratories, Department of Chemistry, Faculty of Science, Ain Shams University, Abbassia, Cairo, 11566 Egypt

**Keywords:** Photobiogas, Zinc oxide, ZnO/TiO_2_ nanocomposite, Capping agent, Hydrothermal synthesis

## Abstract

The photocatalytic degradation (PCD) of ethanol in an inert atmosphere on catalysis was explored in a lab-scale experiment. A morphologically tuned nanostructured controlled ZnO and ZnO/TiO_2_ nanocomposites were synthesized using a simple hydrothermal process under the control of several functionally capping agents marking a milestone in photocatalysis. It is possible that this could be modified to produce photobiogas out of organic dye pollution. The photocatalysts were characterized by the use of X-ray diffraction (XRD), transmission electron microscopy (TEM), Brunauer, Emmett, and Teller (BET) surface area analyzer, Scanning electron microscope (SEM-EDX), and UV–Vis absorption spectroscopy. The photocatalytic activity of the ZnO and ZnO–TiO_2_ composite nanostructures was evaluated for the photodegradation of the Tartrazine (Tr) dye aqueous solution. Where the composite matrix exhibits higher photocatalytic activity than pure ZnO nanomaterials as a result of the synergistic interaction between ZnO and TiO_2_ particles. This is mainly due to its higher surface area than pure ZnO. Moreover, the photocatalytic activity of the tuned nanostructured materials is found to be enhanced by the effect of the capping agent and controlling the morphology. This increase is accompanied by a significant shift in surface shape and band gap energy. According to the findings, the designed morphologies of pure ZnO photocatalyst impacted the formation of photobiogas from the photocatalytic decomposition of ethanol as a model of organic pollutants in wastewater. However, when using composite ZnO/TiO_2_ photocatalysts, the rate of CH_4_ formation is significantly lower than when using ZnO photocatalysts alone. This may be attributed to the synergetic effect between ZnO and TiO_2_ particles which leads to a remarkable reduction in the newly formed band gap energy. This may result in a fast rate of recombination between the photogenerated charge carriers (e^−^ and h^+^).

**Supplementary Information:**

The online version contains supplementary material available at 10.1186/s13065-022-00866-2.

## Introduction

The photocatalytic degradation of organic compounds in an inert atmosphere is the term for producing valuable sound suitable alternative renewable energy. This new emerging technique can be used to create many useful products such as methane production, solar hydrogen, CO_2_ conversion…etc. Photocatalytic reduction of CO_2_ may lead to methane formation [1]. Photocatalytic processes under illumination have gained immense research interest as consideration for future large-scale water purification processes under natural conditions [2, 3]. Additionally, the development of newly emerged photocatalysis for water splitting was reviewed for generating hydrogen, particularly under visible-light irradiation [4, 5].

Although much research on nanostructured semiconductor photocatalysts has resulted in adaptable methods of increasing their photocatalytic efficiency [6, 7], few studies have intensively focused on how much efficiency would be influenced in various morphologies. Reducing particle size is a typical advantage for surface-dependent photocatalysis because it causes a quadratic increase in the specific surface area and hence reactive sites onto the surface of the photocatalyst [8]. The photocatalyst, as well as the light source in the reactor arrangement, are the most imperative and essential components for a successful photocatalytic system [9].

TiO_2_ and ZnO are among the most prospective semiconductor materials that are promising state-of-the-art nanostructured materials that can be used in many applications, and it can be attributed to their interesting physical and optical properties since they are low cost, stable, nontoxic, and ease of availability [10–12]. Likewise, ZnO and TiO_2_ have shown superior photocatalytic capabilities through photoinduced water splitting, as well as self-cleaning glass, for degrading organic compounds [13].

Furthermore, due to their significant band gaps (ZnO3.4 eV and TiO_2_3.2 eV), both ZnO and TiO_2_ work exclusively under UV irradiation and have low efficiency, limiting their practical applications [14–16]. When two semiconductors coupled together provide a novel approach to separating charges efficiently, increasing charge carriers’ lifetimes, and increasing interfacial charge transfer to adsorbed substrates [16–18]. Furthermore, since they have different band gaps, ZnO/TiO_2_ composites can cover a broader solar light spectrum than the pure ones. The opportunity for photocatalytic degradation to produce valuable aliphatic hydrocarbons and hydrogen by generating various organic compounds, counting methanol and ethanol, was previously studied without insight into the morphological tunning effect between TiO_2_ and ZnO [1, 7, 19–21].

Based on the green chemistry approach, the production of photobiogas (CH_4_) from photocatalytic degradation of organic compounds is a promising technology for generating green energy needed for a clean environment [21, 24, 25]. In this work of research, ZnO and ZnO/TiO_2_ nanocomposite structures with different morphologies have been synthesized via a simple and low-cost hydrothermal method that is based on the application of different capping agents (CA), such as cetyltrimethylammonium bromide (CTAB), acetylacetone (ACAC), and poly (4-styrene sulfonic acid) solution (PS). The optical, structural, and morphological characteristics of all the samples have been investigated using UV–Vis spectroscopy, XRD, FTIR, SEM-EDX, and TEM analyses. The photocatalytic activity of the tuned ZnO and ZnO/TiO_2_ nanomaterials has been tested on the photocatalytic degradation of Tartrazine dye, as a model of organic dye pollutants. As well as, the photocatalytic generation of useful hydrocarbons such as biogas (CH_4_) from the photocatalytic decomposition of ethanol under a nitrogen atmosphere over the different prepared ZnO and ZnO/TiO_2_ nanostructures has been investigated. Lab-scale experiments in a designed UV-photoreactor under an N_2_ atmosphere have been used for this purpose.

## Experimental

### Materials

Titanium (IV) Isoperoxide (97%) is obtained from Fluka. Zinc acetate (Zn (CH_3_COO)_2_), acetic acid (96%), Nitric acid, ethanol absolute (99.8%), and hydrochloric acid (HCl) were purchased from Sigma-Aldrich. Sodium hydroxide (NaOH) pellets are obtained from Qualikems. Different materials are used as capping agents during the hydrothermal preparation of the nanomaterials. Such as cetyltrimethylammonium bromide (CTAB), C_19_H_42_N^*^Br from Qaulichem Co. India, (ACAC) From Qaulichem Co. India., and poly (4-styrene sulfonic acid) (PS) from Sigma-Aldrich. Tartrazine (Tr) Tartrazine is a water-soluble dye with the chemical formula: C_16_H_9_N_4_Na_3_O_9_S_2_. Molar mass is 534.36 g mol^−1^. Tartrazine is a chemical that has been known for years to cause water pollution, it is utilized in a wide variety of industrial products, including household cleaners, writing instruments inks, wrapping glues, face paints, and stamp dyes.

### Synthesis of the ZnO nanostructures

An appropriate amount of zinc acetate hydrated (2 g) was dissolved in 100 ml of distilled water to form a transparent solution. A 15 ml sodium hydroxide (NaOH)[5M] was gradually added to the above solution and under vigorous stirring. After the formed Zn (OH)_4_ precipitate was filtered, washed with distilled water, and dried at room temperature. 1 g from the dried powder was added to 60 ml double distilled water in the presence of (2:1) wt/wt capping agent under continuous stirring and sonication for 30 min at room temperature to form a homogeneous suspension. The suspension was then transferred into a 100 ml Teflon-lined stainless-steel autoclave and sealed tightly. Hydrothermal treatment of the slurry was conducted at 160 °C for 48 h. After that, the autoclave was allowed to cool down naturally, and the resultant white product was washed with double distilled water and dried at 60 °C in air. The above procedure was repeated with and without the different capping agents to get the various morphologies from ZnO structures [17, 22, 23]

### Synthesis of ZnO/TiO_2_ nano composite structures with different morphologies

Ti(OH)_4_ powder was obtained by the sol–gel method, as described by Flores et al. [24]. Typically, 10 ml of titanium isoperoxide (Ti(Oi-C_3_H_7_) was added to 100 ml of distilled water under vigorous stirring. Then 10 ml acetic acid was added to the suspension under continuous stirring. A 0.1 m HNO_3_ was added under constant stirring with heating for 4–5 h at 80 °C until transparent sol was obtained. The resulting sol was then dried in an oven at 85 °C to get a powder from Ti (OH)_4_. A 0.5 g from Zn(OH)_4_ powder obtained by the previously described method was added to an equal amount of the Ti (OH)_4_ powder under continuous stirring and sonication for 30 min to form a homogeneous slurry. The suspension was transferred into an 80 ml Teflon-lined autoclave that allowed heat at 160 °C for 48 h. The autoclave was allowed to cool down naturally, and the resultant white product was washed with double distilled water and dried at 60 °C in air. The above procedure was repeated with and without the different capping agents to get the various morphologies from ZnO/TiO_2_ nanocomposites [25]_._

### Characterization

The morphology of produced nanomaterials was identified using a transmission electron microscope (TEM) JEM-2000EX (JEOL, Japan). Scanning electron microscope (SEM) (JCM-7000 NeoScope™ Benchtop with acceleration voltage 10 kV, was evaluated to catch SEM images and to carry out the elemental analysis. For the characterization of the crystal structure and crystallite size, X’pert Philips X-ray diffraction (XRD) with CuK radiation, 40 kV and 30 mA, and scan rate 50 min was used to examine samples. An automatic surface area and pore size analyzer (BELSORP MINI X) was used to determine Specific surface area via adsorption–desorption of N_2_ gas at 77 K. The FT-IR spectra were obtained with a JASCO-FT-IR 6800 spectrometer. The UV–Vis/DR spectra were obtained using a JascoV-650 double-beam spectrophotometer equipped with a photomultiplier tube detector and a single monochromator with BaSO_4_ as a reference. Unicam 8700 spectrometer system over the 190–900-nm wavelength range used to measure UV–vis absorption spectra. The bandgap energies of the catalysts were determined by the Kubelka–Munk function, F(R), and by extrapolating the [F(R)hν]^2^ versus photon energy. The produced gases from the reaction were analyzed using gas chromatography (GC) model SRI 8610C. UV–Vis spectrophotometers A V-630 Jasco UV–Vis Double-beam is used for spectroscopic measurements. It ranges from 190 to 1100 nm with a fixed bandpass of 1.5 nm.

### Photocatalytic activity experiments

The photocatalytic activity of the ZnO and ZnO–TiO_2_ composite nanostructures was evaluated for the photodegradation of Tartrazine (Tr) dye aqueous solution (1 g/l catalyst to 5 × 10^−5^ M dye). After the dark adsorption of the dye solution on the catalyst surface, the slurry was subjected to UV irradiation. At different time intervals, aliquots were withdrawn and centrifuged to remove the catalyst. The clear solution was recorded on a spectrophotometer to evaluate the residual concentration of dye pollutants. The rate kinetics of the degradation reactions was statistically calculated to determine the reaction rate constants for the photodegradation reactions with the different nanostructure materials. Total organic carbon analyzer (TOC), TOC-Analyzer multi-N/C (Analytik Jena AG, Germany), total carbon (TC), and inorganic carbon (TIC) are determined separately. The difference results in the TOC, TOC = TC − TIC. It is mainly used for the TOC determination of aqueous samples with high TOC and low TIC content. TOC is expressed as Parts per Million (ppm or mg/l). During the experiment, samples were taken at the same time intervals and TOC was measured, and the final percentage of organic matter removal at the end of the reaction is calculated as ((TOC_intial_ − TOC_final_)/TOC_initial_)100), and represented as the percentage % TOC removal.

### Photobiogas experiment

The photobiogas production experiments were carried out in UV reactor sets designed for the laboratory, as described by Mozia et al. [21]. Typically, 0.35 g of a catalyst was introduced to 0.35 dm^3^ of ethanol C_2_H_5_OH (1 mol/dm^3^) in the cylindrical glass UV reactor. Nitrogen gas was bubbled into the reactor for at least 30 min to eliminate the dissolved oxygen at the start of the experiment. The photoreaction was then initiated by turning on the UV light that was positioned in the reactor’s middle. Gaseous products were measured using gas chromatography (GC)” SRI 8610C fitted at various time intervals throughout the 5-h experiment. The carrier gas utilized was hydrogen; each test was carried out at least twice to guarantee that the tests could be repeated. The findings were estimated by using the mean values from the two experiments.

## Results and discussion

### Structural and morphological analysis

**Fig. 1:**
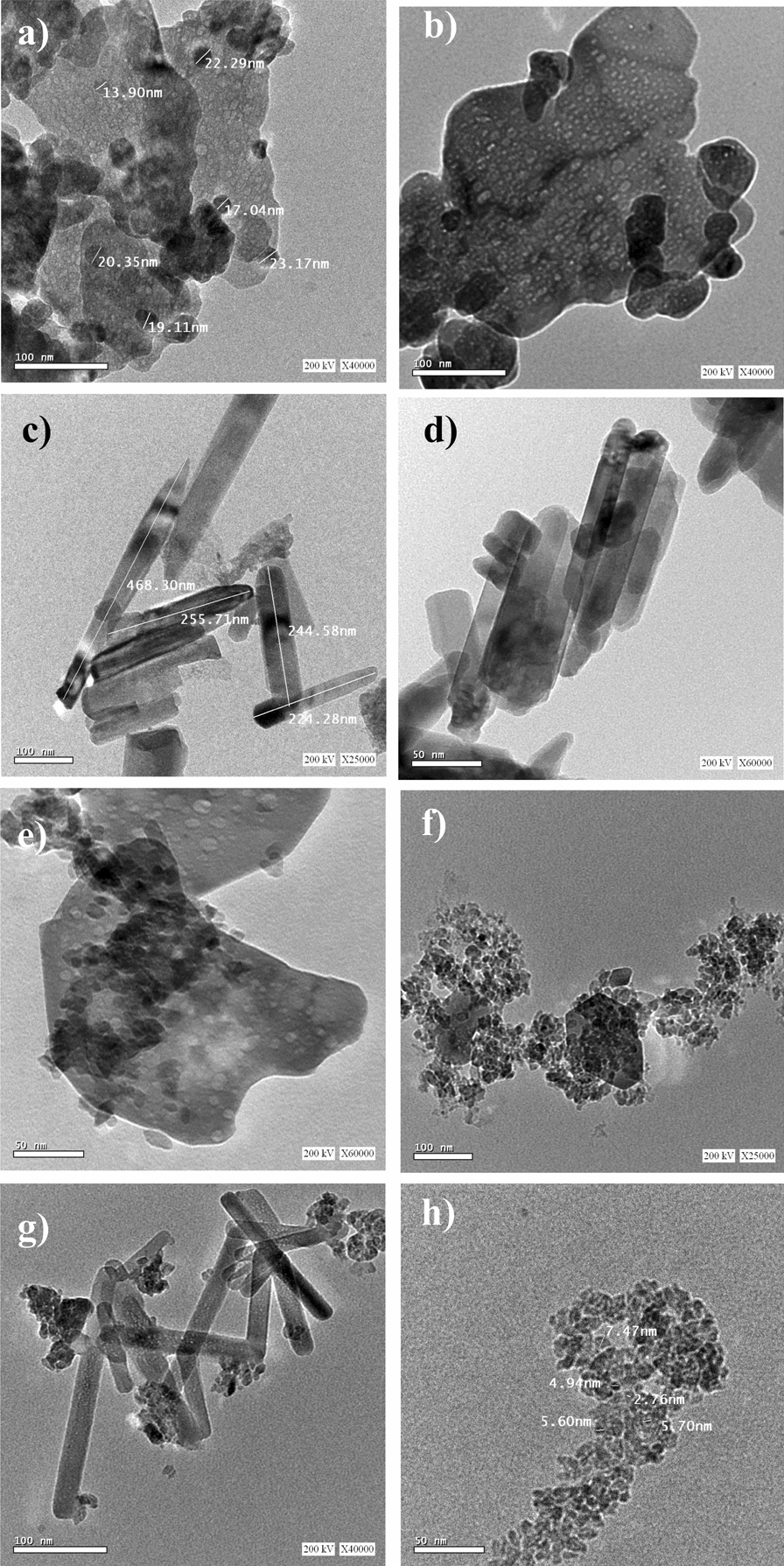
HRTEM images of different ZnO and ZnO/TiO_2_ composite nanostructures hydrothermally synthesized **a** pure ZnO; with (2:1 at. wt. ratio) **b** ZnO (PS); **c** ZnO (CTAB); **d** ZnO (AC AC); **e** pure TiO_2_; **f** ZnO/TiO_2_ (PS); **g** ZnO/TiO_2_ (CTAB); **h** ZnO/TiO_2_ (AC AC); respectively

The morphology of the as-prepared different ZnO structures was observed using Transmission Electron Microscope (TEM) as given in Fig. 1a–d. Figure 1a shows the HRTEM image of ZnO prepared hydrothermally without any capping agent, which reveals that ZnO sheets with the random formation of hexagonal shape from ZnO nanoparticles have been formed during the hydrothermal treatment of Zn(OH)_4_ in pure water. Similar behavior has been observed in the presence of PS as a capping agent (Fig. 1b). However, in the presence of a CTAB capping agent, ZnO with nanorod structure (Fig. 1c) with an average diameter (10–35 nm) and average length (50–160 nm) has been obtained. A large ZnO nanorod structure with an average diameter (30–50 nm) and average length (100–150 nm) has been produced in the presence of an ACAC capping agent (Fig. 1d). In an attempt to discuss and propose the effect of the capping agent on crystal formation growth, it has been suggested that the crystal formation process is divided into two stages nucleation and growth.

The overall reaction for the growth of ZnO nanocrystals may be expressed as follows:

1 $${\text{Zn}}\left( {{\text{CH}}_{{3}} {\text{COO}}} \right)_{{2}} + {\text{ 2NaOH}} \to {\text{Zn}}\left( {{\text{OH}}} \right)_{{2}} + {\text{2CH}}_{{3}} {\text{COONa}}$$

Without a capping agent and under hydrothermal treatment, zinc hydroxide species are transformed into zinc oxide crystals [26].

2 $$\left[ {{\text{Zn}}\left( {{\text{OH}}} \right)_{{4}} } \right]^{{{2} - }} \to {\text{ZnO}} + {\text{H}}_{{2}} {\text{O}} + {\text{2OH}}^{ - }$$

In the presence of a capping agent, the growth of polar inorganic [Zn(OH)_4_]^2−^ nanocrystals is sensitive to the polarity of the capping agent, and its morphologies can be tuned and controlled by the crystal–capping agent interfacial interactions [27]. In such cases, the morphology of ZnO is primarily directed by the polarity of the capping agent. For example, in the presence of CTAB, electrostatic interaction between CTAB (CTA^+^) micelles and zinc species [Zn(OH)_4_]^2−^ will occur, and subsequently, changing of the morphology from nanoparticles to nanorod structure has been performed (Fig. 1c) [28]. In the presence of ACAC, the “O” in the “–C=O–” function group has a negative charge, and “Zn” atoms in the [Zn(OH)_4_]^2−^ particles are positively charged, so an intense interaction will occur between “O” and “Zn” atoms when ACAC was added to the mixed solution in our experiment and ZnO nanorods were produced [29, 30].

From the above results, it can be concluded that the polarity of the capping agent can effectively affect the crystal growth and the morphology of the produced ZnO nanostructures. To investigate the effect of the capping agent concentration on the morphological variation of ZnO nanostructures, different concentrations of used capping agents such as; (3:1 wt/wt). It has been found that the morphologies of the ZnO nanostructures have not changed with changing the concentration of the CA (see Additional file 1) Figure(S1). It means that once the capping agent is added, it will establish a chemical interaction with the surface of the ZnO particle using its functional groups, and further addition of capping molecules has no effect on changing the morphology [31].

The morphology of the obtained ZnO/TiO_2_ composite powders after hydrothermal treatment in the absence and presence of the different capping agents mentioned above was observed by using TEM as given in Fig. 1e–h. From the recorded TEM images of the different samples, it is clear that in all the samples, aggregates of TiO_2_ nanoparticles are formed during the hydrothermal treatment. However, different morphological shapes have been obtained for ZnO in the presence of the other capping agents. Figure 1e shows the HRTEM image of ZnO/TiO_2_ composite prepared hydrothermally without any capping agent, which reveals the formation of ZnO sheets in addition to TiO_2_ nanoparticles with spherical shape. Similar behavior has been observed in the presence of PS capping agents (Fig. 1f). However, in the presence of CTAB capping agents, a mixture of short ZnO nanorods and TiO_2_ nanoparticles has been observed in Fig. 1g. Mixed nanoparticles from ZnO and TiO_2_ that form a porous network structure were formed by hydrothermal treatment in the presence of ACAC as a capping agent (Fig. 1h).

**Fig. 2:**
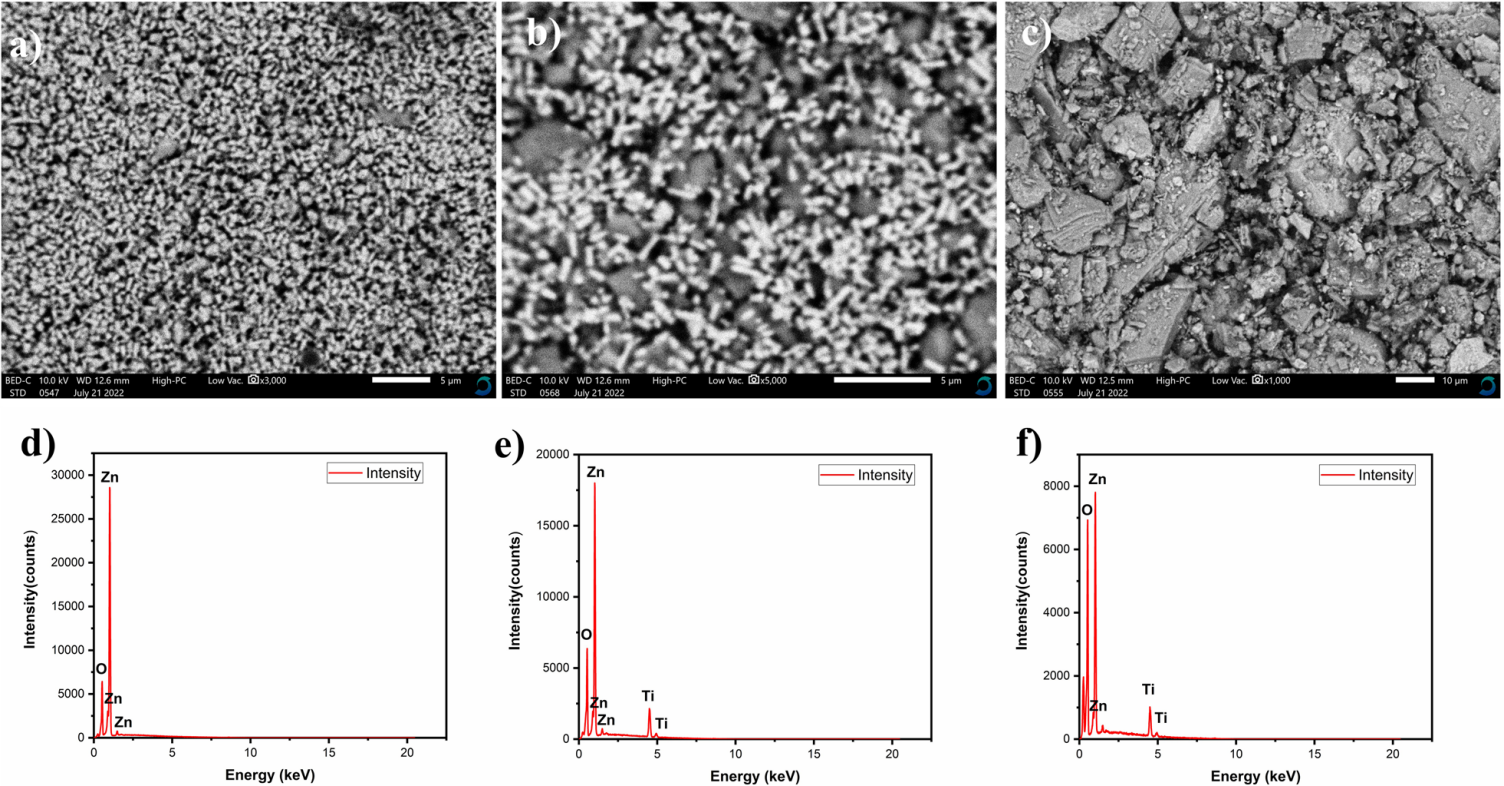
SEM images of **a** ZnO (no CA), **b** ZnO/TiO_2_ (no CA), **c** ZnO/TiO_2_ (AC AC), EDX of **d** ZnO (no CA), **e** ZnO/TiO_2_ (no CA), and **f** ZnO/TiO_2_ (AC AC) respectively

The surface morphology of the prepared samples was investigated by scanning electron microscopy (SEM) and the images acquired are shown in Fig. 2. Highly porous ZnO hexagonal nanosheets with a rough surface are observed (Fig. 2a). These shape analyses are in agreement with TEM results. TheTiO_2_ NPs had a spherical morphology and the surface exhibited a certain degree of roughness which become rougher when coupled with Titanium oxide (Fig. 2b). Sharp ZnO nanorods coupled with TiO_2_ spherical nanoparticles are formed when using AC AC as a capping agent in tuning the surface morphology of the composite (Fig. 2c).

EDX microanalysis was used to confirm the elemental composition of the ZnO/TiO_2_ nanocomposite. Figure 2d) shows clear beaks for Zn and O atoms with their atomic ratios that are given in Table 1. However, the EDX analysis of ZnO/TiO_2_ nanocomposites (Fig. 2e, f), confirms the presence of Zn, Ti, and O atoms in its spectra. No other peaks are observed in the EDX analyses, this confirms the purity of the samples.

**Table 1:** Elemental composition data from EDX measurements

Element	Zn		O		Ti		Total
	Mass%	Atom%	Mass%	Atom%	Mass%	Atom%	
ZnO (no CA)	85.45 ± 0.60	58.97 ± 0.41	14.55 ± 0.21	41.03 ± 0.58	–	–	100.00
ZnO/TiO_2_ (no CA)	55.02 ± 0.49	32.42 ± 0.29	19.59 ± 0.28	47.17 ± 0.66	25.39 ± 0.54	20.41 ± 0.43	100.00
ZnO/TiO_2_ (ACAC)	44.07 ± 0.60	20.26 ± 0.28	35.68 ± 0.47	67.03 ± 0.88	20.24 ± 0.64	12.70 ± 0.40	100.00

**Fig. 3:**
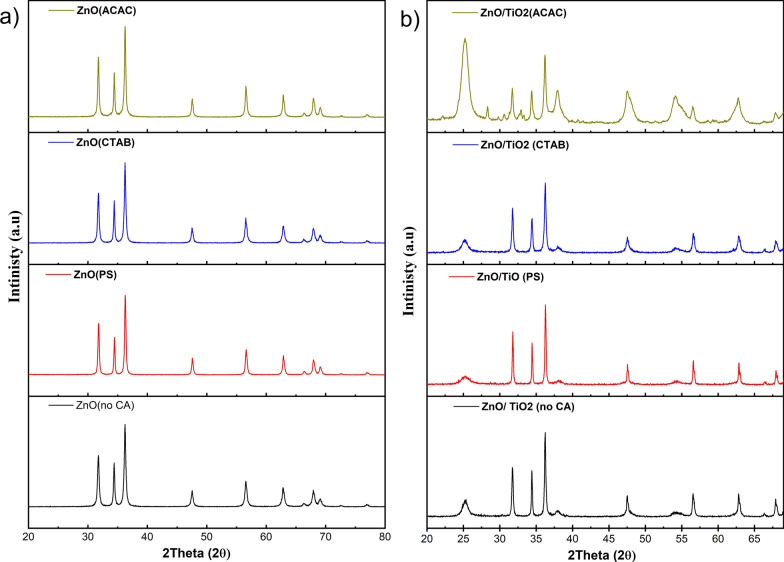
XRD diffraction patterns of **a** ZnO, **b** ZnO/TiO_2_ with different morphological structures

Typical XRD patterns of the as-synthesized ZnO nanostructures with the different capping agents are shown in Fig. 3a. All diffraction peaks can be precisely indexed to the hexagonal ZnO with a wurtzite structure, which is in good agreement with the literature values (JCPDS 36-1451) [32]. No characteristic peaks from other impurities are detected. The XRD patterns of ZnO/TiO_2_ composite structures using various capping agents (2:1 wt/wt), hydrothermally autoclaved at 160 °C for 48 h, are given in Fig. 3b. All the diffraction peaks can be indexed as hexagonal wurtzite-type ZnO (PDF: 96-230-114) and the anatase TiO_2_ (PDF: 6-900-4140). It is evident that the hydrothermal treatment successfully achieved ZnO/TiO_2_ heterojunctions that integrated the wurtzite phase ZnO nanostructures with the anatase TiO_2_ [33–35]. It can be well observed that, for un-capped ZnO/TiO_2_ composite structure (Fig. 3b), the intensities of the main peaks of ZnO and TiO_2_ do not differ from those in the case of pure ZnO or TiO_2_, which means that both oxides are present in the composite as separate phases, and there is no indication for the formation of mixed compound.

Based on Scherrer’s Eq. (3) [36], the average crystallite size of the as-prepared ZnO and ZnO/TiO_2_ nanostructure has been calculated and given in Table 2.

3 $$D=\frac{K\lambda }{\beta cos\theta }$$

where K = 0.9 is a shape factor for spherical particles, λ is the wavelength of the incident radiation (λ = 1.54056 Å), θ is half of the diffraction angle (rad), and β is the line width at half maximum height.

**Table 2:** The average crystal sizes, band gap energies (E_g_), surface area as BET analyses rate constants, and % removal of TOC

Catalyst (Capping agent)	Crystallite size		Surface area		Band gap energy						Rate constant	TOC%
	ZnO	TiO_2_	SBET (m^2^ g^−1^)	V_total_ (cm^3^ g^−1^)	E_g_ (eV)		E_VB_	E_CB_	E_VB_	E_CB_	k (min^−1^)	
					Direct	Indirect	For direct E_g_ (eV)		For indirect E_g_ (eV)			
ZnO	56	–	14.05	0.113	3.162	–	2.78	− 0.38	–	–	0.04 ± 0.003	28.00
ZnO (PS)	30	–	14.99	0.058	3.187	–	2.79	− 0.39	–	–	0.04 ± 0.003	24.02
ZnO (CTAB)	27	–	40.22	0.139	3.194	–	2.80	− 0.40	–	–	0.07 ± 0.004	38.10
ZnO (ACAC)	28	–	47.50	0.113	3.180	–	2.79	− 0.39	–	–	0.08 ± 0.007	69.13
ZnO/TiO_2_	18.00	53.24	113.57	0.12	3.160	3	2.80	− 0.36	2.81	− 0.19	0.06 ± 0.007	31.96
ZnO/TiO_2_ (PS)	13.09	15.70	116.4	0.13	3.177	3.02	2.81	− 0.37	2.82	− 0.20	0.05 ± 0.007	42.19
ZnO/TiO_2_ (CTAB)	12.00	42.01	96.60	0.22	3.192	3.04	2.82	− 0.38	2.83	− 0.21	0.07 ± 0.007	49.32
ZnO/TiO_2_(ACAC)	37.60	43.86	217.08	0.21	3.176	2.84	2.81	− 0.37	2.73	− 0.11	0.09 ± 0.008	90.98

Table 2 presented the surface and structural analysis data of ZnO nanostructures hydrothermally prepared in the presence of the different capping agents. It can be observed that the capping agent used in ZnO growing influences the particle crystallography of both ZnO and ZnO/TiO_2_ nanocomposite powders. As the capping agent changed, the average crystallite sizes changed. At the same time, the average crystallite size of the uncapped ZnO is higher than the capped ZnO nanostructures with the different capping agents used in this study. This may be attributed to the change in growth rate between the various crystallographic planes due to the capping agent’s-controlled morphology during the hydrothermal process [37].

**Fig. 4:**
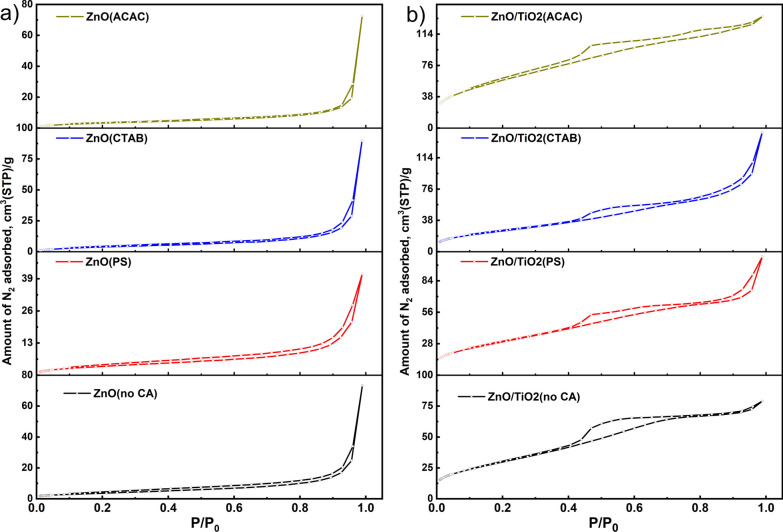
N_2_ adsorption/desorption isotherms of **a** ZnO and **b** ZnO/TiO_2_ with different morphological structures

The N_2_ adsorption/desorption isotherms of the different nanostructures ZnO samples are displayed in Fig. 4a. All the samples can be classified as typical type IV with a well-defined H3 hysteresis loop, indicating the presence of a mesoporous texture in the samples [38]. The capping agent-assisted hydrothermal process significantly increases the surface area and total pore volume (Table 2). More specifically, the surface area increases from 14.05 m^2^ g^−1^ for the uncapped ZnO to the highest value at 47.50 m^2^ g^−1^ in the presence of an ACAC capping agent. It is well known that nanostructures with a larger surface area could work as better photocatalysts. Thus as-prepared different ZnO nanostructures will find a wide variety of applications in nanoscale electronic and optoelectronic devices, as well as photocatalysts [39]. Figure 4b shows the adsorption/desorption isotherms of the different ZnO/TiO_2_ nanostructure products obtained by the hydrothermal method in the presence of the other capping agents. In particular, the surface area increases from 96.60 m^2^ g^−1^ for ZnO/TiO_2_ in the presence of CTAB capping agent to the highest value at 217.08 m^2^ g^−1^ in the presence of ACAC capping agent. It is apparent that changing the capping agent significantly increases the surface area and total pore volume.

Isotherms for adsorption and desorption of ZnO/TiO_2_ composite particles are typically type-IV (IUPAC, 1985), which indicates mesoporous structure. There are also desorption hysteresis loops noted when the relative pressure (P/P_0_) is 0.42 to 0.83. Accordingly of the capillary condensation associated with mesoporous channels in ZnO/TiO_2_ composites, the isotherms are classified as H1, and this type of hysteresis loop is frequently associated with the cylindrical pore geometry and elevated degree of pore uniformity and connectivity in composite catalysts [40]. The composite matrix has a larger surface area than pure ZnO (Table 2), implying that it will have more photocatalytic activity than pure ZnO nanoparticles.

**Fig. 5:**
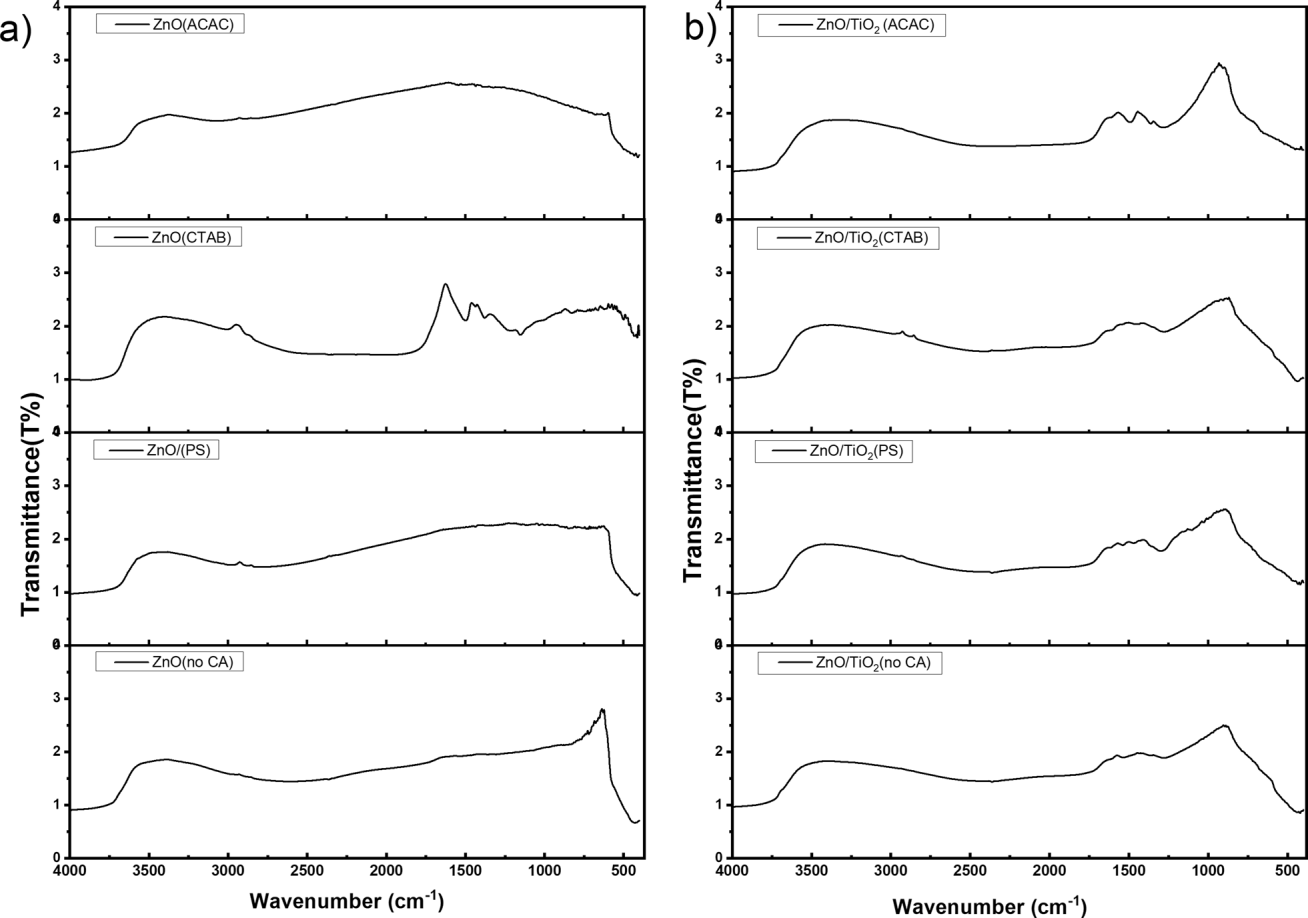
UV–Vis absorption spectra of **a** ZnO and **b** ZnO/TiO_2_ with different morphological structures

The FTIR spectra of ZnO samples are shown in Fig. 5a. The well-resolved full and sharp band centered at around 485 cm^−1^ is ascribed to the stretching vibration of the zinc-oxygen bond in transmission mode, confirming the formation of ZnO [41]. The weak broad band centered at 3490 cm^−1^ is attributed to the stretching vibration of the O–H bond, which may be due to surface adsorbed water molecules or Zn(OH)_2_ [38]. The recorded FTIR spectrum for the uncapped ZnO shows no other characteristic peaks. Extra peaks in (800–2400 cm^−1^) in the samples prepared in the presence of some capping agents are assigned to the various functional groups in the capping agent residues.

The FT-IR spectra of ZnO/TiO_2_ nanocomposites are given in Fig. 5b and compared to observe the vibrational pattern changes of the nanocomposite spectra about those of the parent compounds (Fig. 5a). Nearly all the vibrational information noted in the starting materials was found in the nanocomposite. In all the FTIR spectra, there are two transmittance peaks in the range 500–1000 cm^−1^, which are assigned to the vibrations of Ti–O and Ti–O–Ti framework bonds. This indicates the presence of a TiO_2_ structure in which vibration of the Ti–O bonds is present in the TiO_2_ lattice. Peaks between 2830 and 3000 cm^–1^ are due to the C–H stretching vibration of alkane groups, which may be attributed to the residues of the capping agents after washing [38, 42–44]. The absorption peaks in the 400–700 cm^−1^ could be attributed to the ZnO stretching modes [43–45].

**Fig. 6:**
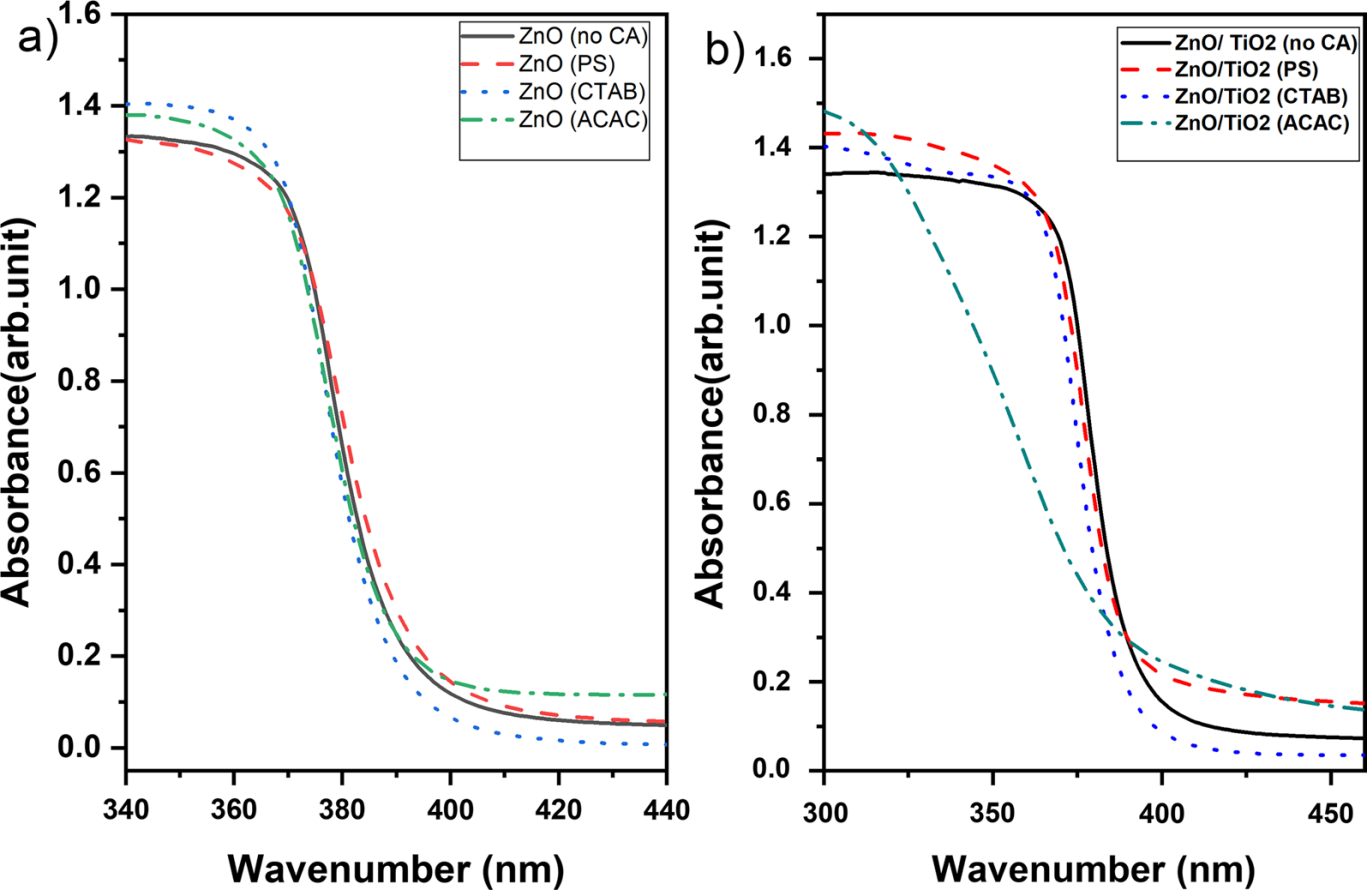
FTIR spectra of **a** ZnO and **b** ZnO/TiO_2_ with different morphological structures

The UV–vis absorption spectra of the uncapped and capped ZnO nanostructures are shown in Fig. 6a. Band gap energy (*E*_*g*_) calculations were carried out employing optical absorption spectra data by using the formula (*αhν)*^2^ ∝ (*hν* − *E*_*g*_), where *α* is the absorption coefficient, *h* the Planck constant, and *ν* the frequency of light, where E_g_ is the band gap energy in eV and λ is the wavelength of nanometers [46]. The allowed direct band gap of differently prepared ZnO nanostructures is calculated and found to be between (3.15–3.22 eV), which is lower than the reported standard band gap energy for ZnO nanoparticles (3.37 eV). Moreover, it can be seen that as the crystal size decreases, an increase in the band gap energy was observed, which could be attributed to the quantum confinement effect [47]. The results are also tabulated in Table 2.

The UV–vis absorption spectra of the uncapped and capped ZnO/TiO_2_ nanostructures are shown in Fig. 6b. The apparent optical bandgap of the ZnO/TiO_2_ composite structures obtained by the hydrothermal synthesis methods in the presence of the different capping agents are calculated and given in Table 2. In general, it has been observed that the band gap energy of the composite structures is lower than that of pure ZnO structures prepared under the same conditions. This could be attributed to the synergistic effect between the conduction band of TiO_2_ and that of ZnO [47, 48].

The band edge values of ZnO and TiO_2_ band edge values can be crucial in separating photoinduced charge pairs. The Butler and Ginley equations were used to estimate the edge potentials of ZnO and TiO_2_ at zero charges:

4 $${\text{E}}_{{{\text{VB}}}} = \chi - {\text{E}}_{{\text{e}}} + 0.{\text{5E}}_{{\text{g}}}$$

5 $${\text{E}}_{{{\text{CB}}}} = {\text{E}}_{{{\text{VB}}}} {-}{\text{E}}_{{\text{g}}}$$

The energy of free electrons on the hydrogen scale and E_VB_ is the VB edge potential, where E_e_ is estimated to be 4.5 eV [48], while χ is the absolute electronegativity of the semiconductor. E_g_ the bandgap energy of the individual photocatalyst. Besides, For ZnO and TiO_2_, electronegativities are about 5.79 [49], and 5.81 eV [50].

As for ZnO, E_VB_ and E_CB_ are calculated and tabulated (Table 2). In comparison with TiO_2_, ZnO has a higher CB and VB. As a result, the electrons at the CB of ZnO will migrate to the CB of TiO_2_ until the Fermi level (E_f_) aligns, whereas the holes at the VB of TiO_2_ will migrate to the VB of ZnO. Thus, the generated electron–hole pairs can be effectively separated. The potentials of the conduction band (CB) and the valence band (VB) of ZnO are slightly more negative than those of TiO_2_. Due to the potential difference between the CB of ZnO and the CB of TiO_2_, the photoexcited electrons in the ZnO–TiO_2_ system will migrate from the CB of ZnO to the CB of TiO_2_.

Under illumination, the holes migrate from the TiO_2_ VB to the ZnO VB. In this way, there is an effective separation of charge carriers that have been induced by absorbed light at the interface of the photocatalyst [50, 51]. As a result of the efficient transfer of photogenerated charge carriers across the interface, the coupling of ZnO with TiO_2_ may exhibit improved photocatalytic activity more surprisingly [52, 53]. Furthermore, by utilizing ZnO as the leading counterpart of the composite, it is possible to tune the morphology of newly generated photocatalysts with increased specific surface areas.

### Photocatalytic degradation of Tr in the presence of ZnO and ZnO/TiO_2_

**Fig. 7:**
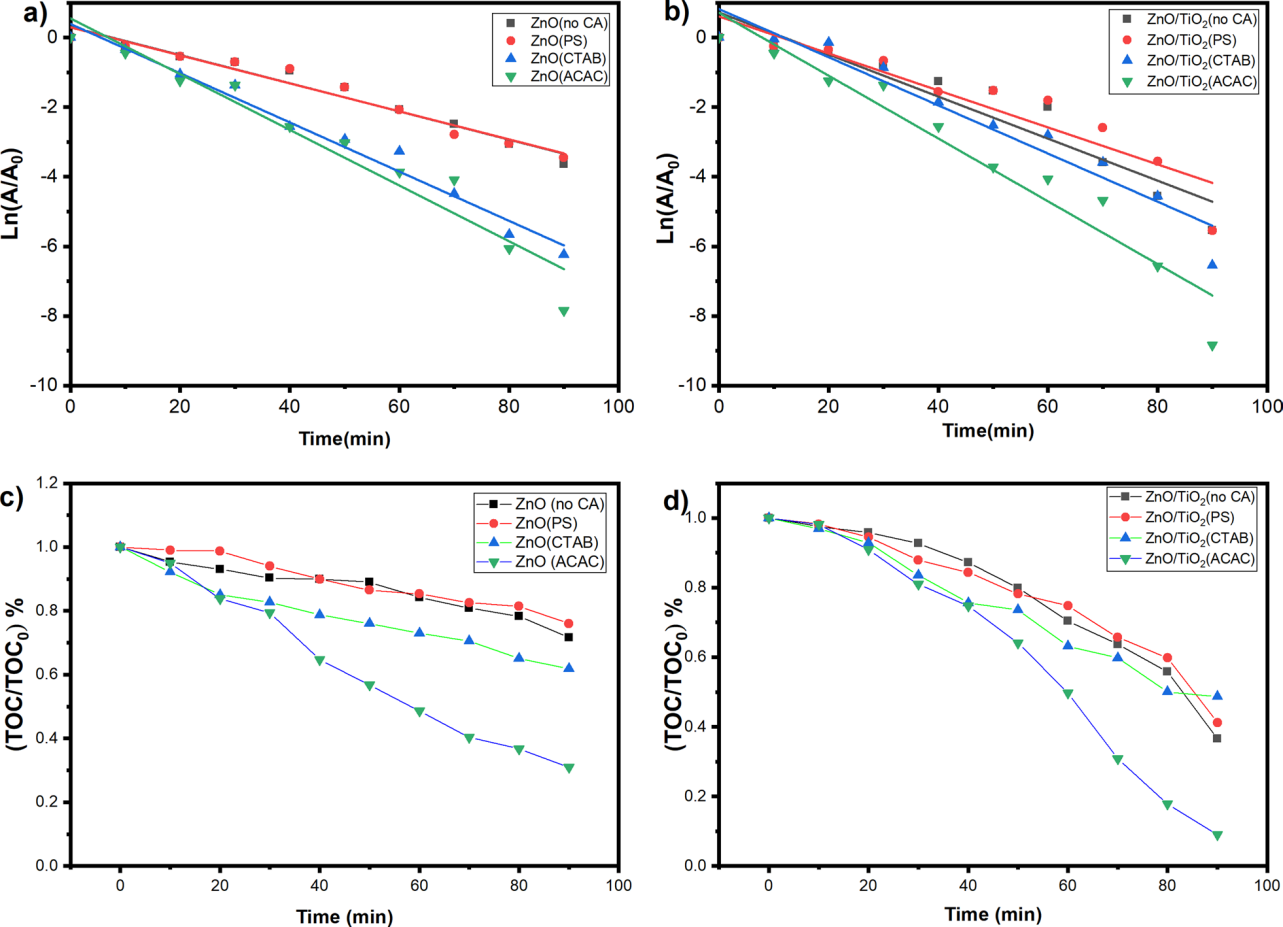
Pseudo-first-order kinetics plots for the degradation of (Tr) by **a** Prepared ZnO nanostructure and **b** ZnO/TiO_2_ composite, Depletion in TOC % removal of (5 × 10^−5^ M) (TR) dye solution under UV-light, **c** Prepared ZnO nanostructure and **d** ZnO/TiO_2_ composite

Figure 7a and b show that the photocatalytic decomposition reactions with suspended ZnO and ZnO/TiO_2_ photocatalysts approximately obey the first-order kinetics. The first order rate constants of the photodegradation reactions under UV illumination have been calculated from the equation; ln(A/A_o_) = − kt, where A is the absorbance of Tr related to its concentration, A_o_ is its absorbance at zero time, and k denotes the overall degradation rate constant.

Hence, the electronic and photocatalytic activity of zinc oxide as a photocatalyst depends strongly on its surface morphology, size, structure, aspect ratio, crystal density, and crystallographic orientation. Applying different capping agents during the hydrothermal treatment process leads to producing various ZnO photocatalysts with different photocatalytic activities depending on these parameters [54, 55]. The above results show that the highest photocatalytic degradation rate obtained with ZnO nanorod structures prepared by hydrothermal treatment using ACAC as a capping agent is a good compactable with the surface area S_BET_ results given in Table 2. Whereas the surface area of the photocatalyst increases, the photocatalytic activity increases. Similarly, it can be seen that The ZnO/TiO_2_ photocatalyst prepared in the presence of ACAC as a capping agent has the highest photocatalytic activity. Findings indicated that the photocatalytic activity of the composite ZnO/TiO_2_ is much higher than that of ZnO photocatalysts prepared under the same conditions. TOC analysis was used to monitor the total organic carbon bound in the (Tr) molecule as an indicator for to complete mineralization of Tr dye. Figure 7c and d show decay in the TOC content during the photodegradation experiments relative to the initial TOC content (TOC_o_). The final TOC decay (TOC/TOC_o_) achieved after only 1.5 h irradiation over the different ZnO and ZnO/TiO_2_ photocatalysts are depicted in Table 2. Significantly, 91% TOC removal has been achieved by using ZnO/TiO_2_ photocatalyst prepared hydrothermally in the presence of ACAC as a capping agent, the obtained results agree with the results of the photocatalytic activity, which shows the effect of tuning the morphology and surface shape on the properties of the prepared materials. In addition, photocatalyst stability is critical in large-scale operations. Therefore, to investigate the stability of ZnO and ZnO/TiO_2_ photocatalysts, recycling experiments of prepared photocatalysts for photocatalytic degradation of Tr under UV irradiation were carried out (see Additional file 1) Figure (S2) Table(S1). The results indicate that the photocatalyst retains photocatalytic solid active and stable under UV irradiation for an extended period.

### Photobiogas production from ethanol

A lab-scale photoreactor has been used for studying the photocatalytic generation of methane (CH_4_) during ethanol decomposition, as a model compound. The different ZnO and ZnO/TiO_2_ nanostructures have been used in these reactions. The photocatalytic decomposition reactions have been done under an N_2_ atmosphere. The main gaseous products detected by GC analysis during these reactions were CO_2_ and CH_4_ gases (Where the gaseous evolve concentrations were expressed in mmol gas/mol ethanol). The results were well reproducible in three replicate measurements.

**Fig. 8:**
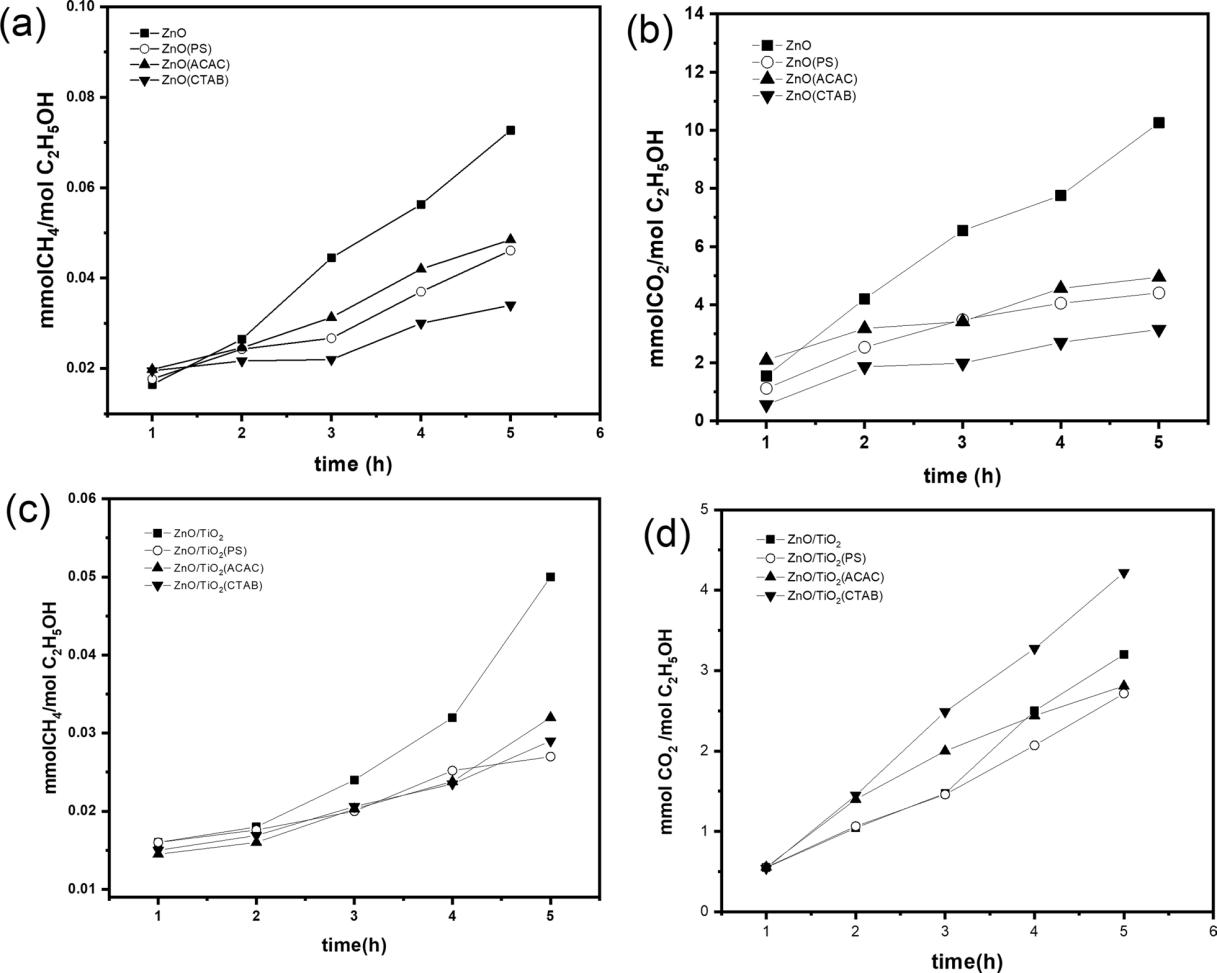
Photocatalytic generation of **a** and **b** CH4 and CO_2_ (mmol/mmol ethanol) gases evolved during photocatalytic decomposition of ethanol using the differently tuned ZnO photocatalysts; **c** and **d** CH4 and CO_2_ (mmol/mmol ethanol) gases evolved during photocatalytic decomposition of ethanol using the different tuned ZnO/TiO_2_ photocatalysts

The progress of CH_4_ and CO_2_ evolution during the photodecomposition reactions of ethanol as a function of UV-irradiation time in the presence of uncapped and capped ZnO nanostructures prepared with PS, ACAC, and CTAB, respectively, are presented in Fig. 8a, b.

From biogas results, it can be noticed that uncapped ZnO has the highest activity and produced the highest amount of CH_4_ production after 5 h of direct UV irradiation. While the lowest amount of CH_4_ evolved was referred to as ZnO capped with CTAB. To explain this behavior that is different from the photocatalytic degradation reactions discussed above, let us consider the possible photocatalytic decomposition mechanism of ethanol in the N_2_ atmosphere given from Eqs. 6 to 13 [56].

Previous studies [57] have reported that ethanol readily dissociates on TiO_2_ to give adsorbed ethoxy species even at room temperature. By a similar mechanism over ZnO photocatalyst, the proposed mechanism can be expressed as follows:

6 $${\text{C}}_{{2}} {\text{H}}_{{5}} {\text{OH}}_{{({\text{g}})}} \to {\text{C}}_{{2}} {\text{H}}_{{5}} {\text{OH}}_{{\left( {\text{a}} \right)}}$$

7 $${\text{C}}_{{2}} {\text{H}}_{{5}} {\text{OH}}_{{({\text{a}})}} \to {\text{C}}_{{2}} {\text{H}}_{{5}} {\text{O}}_{{\left( {\text{a}} \right)}} + {\text{H}}_{{({\text{a}})}}$$

In the first step, dehydrogenation of ethanol to acetaldehyde takes place, which does not need illumination.

Direct UV-illumination of ZnO photocatalyst leads to a promotion of an electron from V_B_ to C_B,_ which results in the formation of the charge carriers e^−^ and h^+^ (Eq. 8)

8 $${\text{ZnO}} + {\text{h}}\upnu \to {\text{e}}^{ - } + {\text{h}}^{ + }$$

The decomposition of the adsorbed ethoxy follows this by the photogenerated electrons into ethoxy species or radicals (Eq. 9)

9 $${\text{C}}_{{2}} {\text{H}}_{{5}} {\text{O}}_{{\left( {\text{a}} \right)}} + {\text{e}}^{ - } \to {\text{C}}_{{2}} {\text{H}}_{{5}} {\text{O}}_{{\left( {\text{a}} \right)}}^{{\updelta - }}$$

This step is followed by the photo-induced decomposition of ethoxy to acetaldehyde and hydrogen:

10 $${\text{C}}_{{2}} {\text{H}}_{{5}} {\text{O}}_{{\left( {\text{a}} \right)}}^{{\updelta - }} \to {\text{CH}}_{{3}} {\text{CHO}}_{{\left( {\text{a}} \right)}}^{{\updelta - }} + {\text{H}}_{{({\text{a}})}}$$

Then photocatalyzed decomposition of acetaldehyde into CH_4_ and CO followed by transformation into CO_2_ [45].

11 $${\text{CH}}_{{3}} {\text{CHO}}_{{\left( {\text{a}} \right)}}^{{\updelta - }} \to {\text{CH}}_{{4}} + {\text{CO}}^{{\updelta - }}_{{\left( {\text{a}} \right)}}$$

12 $${\text{CO}}^{{\updelta - }}_{{\left( {\text{a}} \right)}} + {\text{H}}_{{2}} {\text{O}} \to {\text{H}}_{{2}} + {\text{CO}}_{{2}}$$

The overall reaction of ethanol decomposition can be written as follows [58]:

13 $${\mathrm{C}}_{2}{\mathrm{H}}_{5}\mathrm{OH}+{\mathrm{H}}_{2}\mathrm{O}\to {\mathrm{CO}}_{2}+2{\mathrm{H}}_{2} +{\mathrm{CH}}_{4}$$

Based on the above reaction mechanism, it can be concluded that methane or biogas can be produced by the photocatalyzed decomposition of ethanol over ZnO photocatalyst by UV-illumination under an N_2_ atmosphere. Moreover, it can be well recognized that the primary step in the production of biogas is the formation of charge carriers over the surface of the photocatalyst, which leads to the formation of negatively charged electrons (e^−^) on the C_B_ of the catalyst surface.

In the presence of the different ZnO nanostructures mentioned above, it can be well recognized that uncapped ZnO gives the highest yield of biogas (CH_4_). This can be attributed to the decrease in its band gap energies concerning the others. Given the change in the band gap of the differently prepared ZnO nanostructures (1), it may be concluded that the extent of photolysis of ethanol on ZnO is markedly enhanced by the narrowing of the band gap of the prepared ZnO. This can be explained by the prevention of electron–hole recombination [58, 59].

For comparison, the photocatalytic decomposition of ethanol has also been tested over ZnO/TiO_2_ nanostructured materials prepared under the same conditions.

Figure 8c and d present the progress of CH_4_ and CO_2_ evolution during the photocatalytic decomposition reactions of ethanol as a function of UV-irradiation time in uncapped and capped ZnO/TiO_2_ nanostructures prepared with PS, ACAC, and CTAB, respectively.

Similar behavior has been observed in the case of the application of ZnO/TiO_2_ nanostructures in the photocatalytic decomposition of ethanol to produce biogas (CH_4_), as the highest yield for biogas was obtained by uncapped ZnO/TiO_2_. However, it is well noticed that, in general, the rate of CH_4_ production over ZnO photocatalysts is much higher than that of composite ZnO/TiO_2_ photocatalysts. This may be attributed to the synergetic effect between ZnO and TiO_2_ particles, which remarkably reduces the newly formed band gap energy (Table 1). This may result in a fast recombination rate between the photogenerated charge carriers (e^−^ and h^+^) [59]. This can be due to the advantageous effects of their high porosity.

Therefore, current work investigates and employs the ZnO/TiO_2_ nanocomposite photocatalyst more efficiently for charge carrier and surface morphology activator. Thus, tuned controllable attaching TiO_2_ with well-organized ZnO nanostructures will generate perfect synergy, providing superior photocatalytic performance. By morphologically tuning the carriers, the separation efficiency of photoinduced charge carriers will be increased, and the light-harvesting capacity will be enhanced remarkably. Hence, the balanced synergy will increase the efficiency of the designed photocatalyst.

## Conclusions

In conclusion, structurally controlled ZnO/TiO_2_ composite structures have been synthesized via a low-temperature hydrothermal method (160 °C) with the aid of different capping agents. Different ZnO/TiO_2_ nanocomposites with variant tunable morphology and significantly high surface areas have been synthesized. This implies that the coupling between TiO_2_ and ZnO semiconductor materials improves the general properties of the obtained semiconductor nanomaterials. The control of the synthesis of ZnO/TiO_2_ nanocomposite for customizing the crystallinity, surface porosity, and morphology of the photocatalysts also provides significant opportunities to have a crucial influence on photoactivity. Production of valuable hydrocarbons such as biogas (CH_4_) compounds as a result of the photocatalytic degradation of organic pollutants based on tuned morphological structured nanomaterial from ZnO and ZnO/TiO_2_ could be a promising technology for the generation of green energy.

## Supplementary Information

**Additional file 1. Figure (S1):** HRTEM images of different ZnO and ZnO/TiO_2_ composite synthesized (a) ZnO(ACAC) 3:1wt: wt (b) ZnO(ACAC) 2:1 3:1wt: wt. and Stability of Photocatalyst experiment.** Figure (S2)**: Under UV irradiation, the photocatalyst undergoes cycling for the degradation of (Tr)solution. B) After-use powder X-ray diffractograms For ZnO and ZnO (ACAC) C) After-use powder X-ray diffractograms For ZnO/TiO_2_ and ZnO/TiO_2_ (ACAC) respectively.** Table (S1)**: Rate constants recycling experiments of prepared photocatalysts.

## Data Availability

All data are fully available without restriction. All the data obtained investigated in this research are accessible to the corresponding author (Omar Mbrouk).
