# Supplementary material for: Stimulated generation of photobiogas by morphologically tuned nanostructured ZnO and ZnO/TiO2
Source: BMC Chem. 2022 Oct 3;16(1):74. doi: 10.1186/s13065-022-00866-2 (PMC9531480; doi:10.1186/s13065-022-00866-2)
Supplement: Supplementary file 1 — Additional file 1. Figure (S1): HRTEM images of different ZnO and ZnO/TiO2 composite synthesized (a) ZnO(ACAC) 3:1wt: wt (b) ZnO(ACAC) 2:1 3:1wt: wt. and Stability of Photocatalyst experiment. Figure (S2): Under UV irradiation, the photocatalyst undergoes cycling for the degradation of (Tr)solution. B) After-use powder X-ray diffractograms For ZnO and ZnO (ACAC) C) After-use powder X-ray diffractograms For ZnO/TiO2 and ZnO/TiO2 (ACAC) respectively. Table (S1): Rate constants recycling experiments of prepared photocatalysts. [file 13065_2022_866_MOESM1_ESM.docx]

**Additional information files**


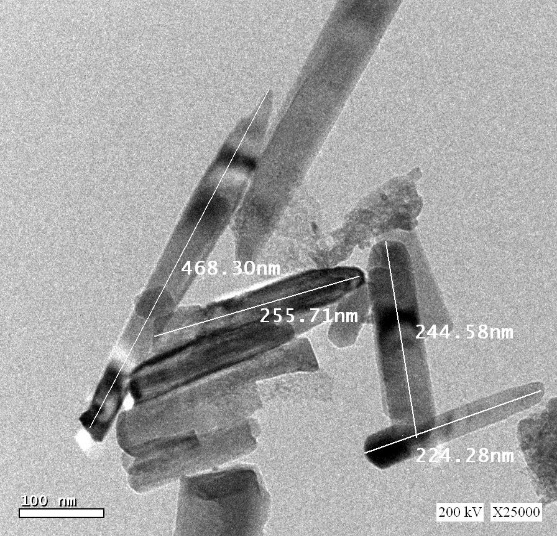

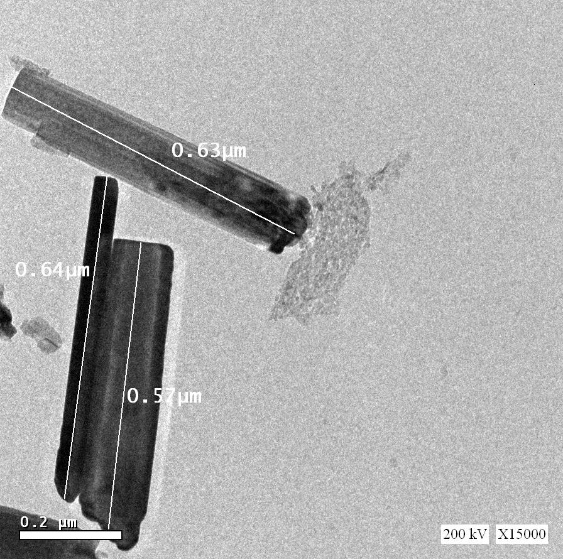


**Figure(S1):** HRTEM images of different ZnO and ZnO/TiO_2_ composite synthesized(a) ZnO(ACAC) 3:1wt:wt (b) ZnO(ACAC) 2:13:1wt:wt.

**Stability of Photocatalyst experiment**

In addition, photocatalyst stability is critical in large-scale operations. Therefore, to investigate the stability of ZnO and ZnO/TiO_2_ photocatalysts, recycling experiments of prepared photocatalysts for photocatalytic degradation of Tr under UV irradiation were carried out see table (S1). The findings were validated by performing powder XRD tests on the photocatalyst after usage, and the diffractograms obtained before and after were compared, as shown in Fig. S1. After each cycle, the photocatalyst was centrifuged, rinsed with distilled water, acetone and ethanol, and dried in an oven at 80 °C. The sample was then reused for subsequent degradation. As can be seen, the rate constant after four cycles, changed of Tr decreased from 0.04, 0.08, 0.06, and 0.09 min^-1^ to 0.35,0.069,0.054 and 0.08 min^-1^for ZnO (no CA), ZnO(ACAC), ZnO/TiO_2_(no CA) and ZnO/TiO_2_(AC AC) respectively. Due to the unavoidable loss of photocatalysts during the cycle operations, the photocatalytic activity only slightly reduces. As a result, the photocatalyst retains strong photocatalytic activity and stability under UV irradiation for an extended period.





c)

b)

a)

Figure (S2): Under UV irradiation, the photocatalyst undergoes cycling for the degradation of (Tr)solution. B) After-use powder X-ray diffractograms For ZnO and ZnO (ACAC) C) After-use powder X-ray diffractograms For ZnO/TiO_2_ and ZnO/TiO_2_ (ACAC) respectively.

Table(S1): Rate constants recycling experiments of prepared photocatalysts

|  | 1^st^ run | 2^nd^ run | 3^rd^ run | 4^th^ run |
| --- | --- | --- | --- | --- |
| ZnO (no CA) | 0.04 | 0.038 | 0.035 | 0.035 |
| ZnO (AC AC) | 0.08 | 0.075 | 0.07 | 0.069 |
| ZnO/TiO_2_(no CA) | 0.06 | 0.058 | 0.056 | 0.054 |
| ZnO/TiO_2_(AC AC) | 0.09 | 0.087 | 0.084 | 0.08 |
